# WHO Essential Medicines Policies and Use in Developing and Transitional Countries: An Analysis of Reported Policy Implementation and Medicines Use Surveys

**DOI:** 10.1371/journal.pmed.1001724

**Published:** 2014-09-16

**Authors:** Kathleen Anne Holloway, David Henry

**Affiliations:** 1 Regional Office for South-East Asia, World Health Organization, New Delhi, India; 2 Institute for Clinical Evaluative Sciences, Toronto, Ontario, Canada; 3 Dalla Lana School of Public Health, University of Toronto, Toronto, Ontario, Canada; 4 Institute for Health Policy Management and Evaluation, University of Toronto, Toronto, Ontario, Canada; Columbia University Mailman School of Public Health, United States of America

## Abstract

**Background:**

Suboptimal medicine use is a global public health problem. For 35 years the World Health Organization (WHO) has promoted essential medicines policies to improve quality use of medicines (QUM), but evidence of their effectiveness is lacking, and uptake by countries remains low. Our objective was to determine whether WHO essential medicines policies are associated with better QUM.

**Methods and Findings:**

We compared results from independently conducted medicines use surveys in countries that did versus did not report implementation of WHO essential medicines policies. We extracted survey data on ten validated QUM indicators and 36 self-reported policy implementation variables from WHO databases for 2002–2008. We calculated the average difference (as percent) for the QUM indicators between countries reporting versus not reporting implementation of specific policies. Policies associated with positive effects were included in a regression of a composite QUM score on total numbers of implemented policies. Data were available for 56 countries. Twenty-seven policies were associated with better use of at least two percentage points. Eighteen policies were associated with significantly better use (unadjusted *p*<0.05), of which four were associated with positive differences of 10% or more: undergraduate training of doctors in standard treatment guidelines, undergraduate training of nurses in standard treatment guidelines, the ministry of health having a unit promoting rational use of medicines, and provision of essential medicines free at point of care to all patients. In regression analyses national wealth was positively associated with the composite QUM score and the number of policies reported as being implemented in that country. There was a positive correlation between the number of policies (out of the 27 policies with an effect size of 2% or more) that countries reported implementing and the composite QUM score (*r* = 0.39, 95% CI 0.14 to 0.59, *p = *0.003). This correlation weakened but remained significant after inclusion of national wealth in multiple linear regression analyses. Multiple policies were more strongly associated with the QUM score in the 28 countries with gross national income per capita below the median value (US$2,333) (*r = *0.43, 95% CI 0.06 to 0.69, *p = *0.023) than in the 28 countries with values above the median (*r = *0.22, 95% CI −0.15 to 0.56, *p = *0.261). The main limitations of the study are the reliance on self-report of policy implementation and measures of medicine use from small surveys. While the data can be used to explore the association of essential medicines policies with medicine use, they cannot be used to compare or benchmark individual country performance.

**Conclusions:**

WHO essential medicines policies are associated with improved QUM, particularly in low-income countries.

*Please see later in the article for the Editors' Summary*

## Abstract 2

Kathleen Holloway and David Henry evaluate whether countries that report having implemented WHO essential medicines policies have higher quality use of medicines.

*Please see later in the article for the Editors' Summary*

## Editors' Summary

**Background:**

The widespread availability of effective medicines, particularly those used to treat infectious diseases, has been largely responsible for a doubling in the average global life expectancy over the past century. However, the suboptimal use (overuse and underuse) of medicines is an ongoing global public health problem. The unnecessary use of medicines (for example, the use of antibiotics for sore throats caused by viruses) needlessly consumes scarce resources and has undesirable effects such as encouraging the emergence of antibiotic resistance. Conversely, underuse deprives people of the undisputed benefits of many medicines. Since 1977, to help optimize medicine use, the World Health Organization (WHO) has advocated the concept of “essential medicines” and has developed policies to promote the quality use of medicines (QUM). Essential medicines are drugs that satisfy the priority needs of the human population and that should always be available to communities in adequate amounts of assured quality, in the appropriate dosage forms, and at an affordable price. Policies designed to promote QUM include recommendations that medicines should be free at the point of care and that all health care professionals should be educated about the WHO list of essential medicines (which is revised every two years) throughout their careers.

**Why Was This Study Done?:**

Surveys of WHO member countries undertaken in 2003 and 2007 suggest that the implementation of WHO policies designed to promote QUM is patchy. Moreover, little is known about whether these policies are effective, particularly in middle- and low-income countries. For most of these countries, it is not known whether any of the policies affect validated QUM indicators such as the percentage of patients prescribed antibiotics (a lower percentage indicates better use of medicines) or the percentage of patients treated in compliance with national treatment guidelines (a higher percentage indicates better use of medicines). Here, the researchers analyze data from policy implementation questionnaires and medicine use surveys to determine whether implementation of WHO essential medicines policies is associated with improved QUM in low- and middle-income countries.

**What Did the Researchers Do and Find?:**

The researchers extracted data on ten validated QUM indicators and on implementation of 36 policy variables from WHO databases for 2002–2008 and compared the average differences for the QUM indicators between low- and middle-income countries that did versus did not report implementation of specific WHO policies for QUM. Among 56 countries for which data were available, 27 policies were associated with improved QUM. Four policies were particularly effective, namely, doctors' undergraduate training in standard treatment guidelines, nurses' undergraduate training in standard treatment guidelines, the existence of a ministry of health department promoting the rational use of medicines, and the provision of essential medicines free to all patients at point of care. The researchers also analyzed correlations between how many of the 27 effective policies were implemented in a country and a composite QUM score. As national wealth increased, both the composite QUM score of a country and the reported number of policies implemented by the country increased. There was also a positive correlation between the numbers of policies that countries reported implementing and their composite QUM score. Finally, the implementation of multiple policies was more strongly associated with the composite QUM score in countries with a gross national income per capita below the average for the study countries than in countries with a gross national income above the average.

**What Do These Findings Mean?:**

These findings show that between 2002 and 2008, the reported implementation of WHO essential medicines policies was associated with better QUM across low- and middle-income countries. These findings also reveal a positive correlation between the number of policies that countries report implementing and their QUM. Notably, this correlation was strongest in the countries with the lowest per capita national wealth levels, which underscores the importance of essential medicines policies in low-income countries. Because of the nature of the data available to the researchers, these findings do not show that the implementation of WHO policies actually causes improvements in QUM. Moreover, the age of the data, the reliance on self-report of policy implementation, and the small sample sizes of the medicine use surveys may all have introduced some inaccuracies into these findings. Nevertheless, overall, these findings suggest that WHO should continue to develop its medicine policies and to collect data on medicine use as part of its core functions.

**Additional Information:**

Please access these websites via the online version of this summary at http://dx.doi.org/10.1371/journal.pmed.1001724.

- The World Health Organization provides information about essential medicines; its latest lists of essential medicines are available on the Internet; information about WHO policies to improve the quality use of medicines is also available (in several languages)
- The International Network for the Rational Use of Drugs designs, tests, and disseminates effective strategies to improve the way drugs are prescribed, dispensed, and used, particularly in resource-poor countries
- The essentialdrugs.org website helps health care professionals, researchers, and policy makers obtain and discuss current information on essential drugs, policy, program activities, education, and training (available in several languages); the website is run by Satellife, which aims to use technology to connect health workers in resource-limited countries to each other and to up-to-date clinical and public health content

## Introduction

Suboptimal use of medicines (over- and underuse) is a global public health problem with serious consequences [1]–[3]. Unnecessary use consumes scarce resources and has adverse effects, including antibiotic resistance [4]–[6] and avoidable adverse drug events, hospitalisation, and death [7]–[9]. Underuse deprives people of the undisputed benefits of many essential medicines [1]–[3].

Since 1977, the World Health Organization (WHO) has advocated the “essential medicines” concept and has developed a range of policies to promote quality use of medicines (QUM) [10],[11]. However, WHO surveys of member countries in 2003 and 2007 showed that fewer than half of responding countries reported implementing many of the recommended policies [12],[13].

Policy implementation increases with country income level [12],[13]. QUM also improves with income level. For instance, adherence to guidelines is greater, and misuse of antibiotics less, in middle-income compared with low-income countries [3],[14],[15]. Coordinated medicine policy implementation in some high-income countries in Europe has resulted in improved antibiotic use [16]–[18]. However, there is little evidence of policy effectiveness in developing countries [19], and associated doubts may contribute to low uptake.

Our aim was to determine whether public sector medicine use is better in low- and middle-income countries that have implemented essential medicines policies than in those that have not. Our hypotheses were that (1) countries implementing essential medicines policies have better QUM than those that do not, (2) policies vary in their effectiveness, and (3) those countries implementing a larger number of effective policies have better QUM than those implementing fewer policies.

## Methods

Most WHO member countries do not publish national data on QUM and policy implementation, nor do they collect time series data that enable longitudinal analyses of the impacts of policies on QUM. Consequently, we used the results of survey data to derive appropriate measures and compare QUM in countries that did versus did not report implementing certain medicine policies to the WHO. In addition, we correlated the numbers of key policies that have been reported by different countries with a composite measure of QUM.

### Information Sources

In order to monitor the impact of essential medicines policies, the WHO, in collaboration with the Boston University/Harvard University WHO Collaborating Center in Pharmaceutical Policy, collects information on medicine use [3],[14],[15] and reported medicine policy implementation [12],[13]. Because of lack of data on medicine use in the private health care sector, information was limited to the public sector.

#### Medicine use database

Studies reporting quantitative measures of medicine use during 1990–2009 in primary health care in any region/country other than North America, Western Europe, Australia, New Zealand, and Japan [3],[14] were identified by systematic searches in PubMed, searches on institutional web sites, and hand searching of 50 journals using standard search terms such as “drug use”, “drug utilization”, “prescribing”, and “developing countries”. The WHO refers to these countries as “developing or transitional”, and the majority are of low- or middle-income status. Only studies reporting standardised measures of QUM [20],[21] were used. Data on medicine use and details of study setting and methodology were extracted from the articles/reports and entered into a Microsoft Access database. The 102 explicitly defined data fields covered setting (e.g., type of facility and prescriber, sector, year of survey), survey methods, intervention details (if any), and measures of medicine use, using a set of predetermined rules [3]. Data from multiple countries or multiple settings (e.g., different facility or prescriber type) described in the same article were entered as separate records, and data from the same study reported in multiple articles were entered as a single record. All articles/reports were read by two persons, and all data entry for each field agreed to by the same two persons (including K. A. H.). Distributions of key variables were scrutinised to assess data entry accuracy prior to analysis.

Nine hundred studies from 104 countries are entered into the database for the period 1 January 1990–31 December 2009. Only 26% of studies addressed medicine use in the private sector [14]. Analyses of the database have been published by the WHO (for 1990–2006, including actual details of data) [3] and in research journals (for 1990–2009) [14],[15]. For the purposes of this study, quantitative data on public sector medicine use by country for surveys undertaken during 2002–2008 were extracted. Only surveys using validated measures of QUM recommended by the International Network for the Rational Use of Drugs (INRUD) and the Integrated Management of Childhood Illness (IMCI) programmes [20],[21] and estimated from at least 600 prescriptions or three or more facilities were included.

#### Database on reported policy implementation

The WHO collects data on reported pharmaceutical policy implementation by sending a questionnaire to the ministries of health of all member states every 4 y. The questionnaire requests information in a standard format [22] on all aspects of pharmaceutical policy implementation, regulation, and practices. The policy questionnaire was developed by the WHO in 2002 in collaboration with the Boston University/Harvard University WHO Collaborating Center in Pharmaceutical Policy, using experience gained from a much smaller pilot survey done in 1999; the questionnaire was further refined during an expert meeting in 2006 [23]. The questionnaires used in this study were sent in 2003 and 2007 in electronic (email) and paper (post) format to all 192 WHO member states through official WHO channels, and follow-up was done by WHO staff. Countries were asked to nominate a government staff member in the ministry of health to coordinate completion of different sections of the questionnaire by the relevant government agencies/departments or expert groups. All completed questionnaires were processed centrally and quality checked, and erroneous or inconsistent data were excluded. Response parameters were set for each question, and any responses falling outside these parameters were verified (going back to the country respondents if necessary) before being manually entered into the database. Data accuracy was assessed by cross-checking and triangulation with data from other sources. Data analysis was done in Microsoft Excel; results for the 2003 survey were published by the WHO in 2006 [12], and those of the 2007 survey in 2010 [13]. Out of 192 WHO member states, 156 countries in 2003 and 146 countries in 2007 responded to the questionnaire, although no country completed responses to all questions (the questionnaire comprises more than 200 questions, including core and sub-questions). For the purposes of this study, data on implementation of policies hypothesized to affect QUM were extracted from responses to questions asked exactly the same way in the questionnaires sent to the ministries of health in 2003 and 2007 [12],[13]. The WHO sent the policy questionnaire to ministries of health in 2011 [24], but these policy data cannot be used to assess impact on medicines use because medicines use surveys done in 2011–2012 have not yet been fully reported.

### Merged Dataset of Quality Use of Medicines and Reported Policy Implementation

We formed one dataset containing all the exposure and outcome data—policies reported as implemented and QUM measures for the period 2002–2008. Only one set of policy and medicine use data for each country was included. We included medicines use surveys from 2002 to 2008 (i.e., from 1 y before the 2003 policy survey to 1 y after the 2007 policy survey) in order to increase the sample size of countries with medicine use data (increase of eight countries, with 377 extra medicine use measurements). In doing this, we assumed that most policies would have been the same within 1 y of the policy survey. For countries where more than one survey was done using the same medicine use indicator during 2002–2008, an average was taken across the surveys to increase the sample size and improve generalisability of the results, giving equal weight to each survey. Where policy information was available for both 2003 and 2007, and found to differ, the policy information chosen was that which was within 1 y of the medicines use survey dates; otherwise, the data were excluded from analysis.

### Derivation of Variables for Analysis

#### Policy variables

The policy variables (Table 1) were derived from country responses to the WHO questionnaires seeking information on implementation of specific medicine policies (see above) [22]. Most were yes/no categorical variables. Where variables had a graded response, we categorised them as yes/no variables (details are provided in Table 1). Fifty-two relevant policies were identified [12],[13]. Because of overlap we did not include them all. For example, the questions on implementation of drug and therapeutics committees ask separately whether they exist in half or more of referral hospitals, general hospitals, and provinces; for the purposes of this study, only the policies on drug and therapeutics committees in general hospitals and provinces were examined. This process of eliminating overlapping questions resulted in 36 distinct policies. Although there was still overlap between them, we erred on the side of inclusion, since only a limited number of countries reported data for all policies. Table 1 shows the selected policy variables plus those excluded (generally because of duplication or irrelevance to prescribing in the public sector).

**Table 1:** Medicine policy variables with reasons for inclusion or exclusion in the analysis.

Policies Hypothesized to Improve QUM*	Inclusion/Exclusion from Analysis (with Reasons)
**NMP and monitoring QUM**	
NMP document	Excluded since there are two other variables indicative of NMP implementation
NMP implementation plan	Included
NMP integrated into national health plan	Included
National strategy to contain antimicrobial resistance	Included
National prescribing audit in the last 5 y	Included
Prescription audit in the last 2 y	Excluded because insufficient numbers of countries responded
**Educational policies**	
Undergraduate training of doctors on the essential medicines list	Included
Undergraduate training of nurses on the essential medicines list	Included
Undergraduate training of pharmacists on the essential medicines list	Excluded because there were insufficient data on pharmacist prescribing, which occurs primarily in the private sector
Undergraduate training of pharmacy assistants on the essential medicines list	Excluded because there were insufficient data on pharmacist assistant prescribing, which occurs primarily in the private sector
Undergraduate training of paramedics on the essential medicines list	Included
Undergraduate training of doctors on standard treatment guidelines	Included
Undergraduate training of nurses on standard treatment guidelines	Included
Undergraduate training of pharmacists on standard treatment guidelines	Excluded because there were insufficient data on pharmacist prescribing, which occurs primarily in the private sector
Undergraduate training of pharmacy assistants on standard treatment guidelines	Excluded because there were insufficient data on pharmacy assistant prescribing, which occurs primarily in the private sector
Undergraduate training of paramedics on standard treatment guidelines	Included
Continuing medical education of doctors	Included
Continuing medical education of nurses and paramedics	Included
Continuing medical education of pharmacists	Excluded because there were insufficient data on pharmacist prescribing
Continuing medical education of pharmacy assistants	Excluded because there were insufficient data on pharmacy assistant prescribing
Public education on antibiotics in last 2 y	Included
Public education on injections in last 2 y	Included
**Managerial policies**	
National essential medicines list updated in the last 5 y	Excluded because insufficient numbers of countries responded to make a comparison (last 2 y used instead)
National essential medicines list updated in the last 2 y	Included
Public insurance drug coverage limited to national essential medicines list	Included
National standard treatment guidelines updated in the last 5 y	Excluded because insufficient numbers of countries responded to make a comparison (last 2 y used instead)
National standard treatment guidelines updated in the last 2 y	Included
National formulary updated in the last 5 y	Included
Generic prescribing in public sector	Included
Generic substitution in public sector	Included
**Economic policies**	
Some drugs covered by public health insurance	Included but graded response adapted to yes/no response as follows: all/some population covered = yes; none covered = no
Coverage of some of the population by public health insurance	Included but graded response adapted to yes/no response as follows: all/some population covered = yes; none covered = no
Dispensing prescribers in public sector	Excluded because of similarity to “No revenue for prescribers from drug sales” and because this question did not exist in 2003 questionnaire
No revenue for prescribers from drug sales	Included but graded response adapted to yes/no response as follows: never used = yes; always/occasionally used = no
Provision of essential medicines free at the point of care to all patients	Included
Provision of essential medicines free at the point of care to patients <5 y of age	Included
Provision of essential medicines free at the point of care to pregnant women	Excluded because of similarity to other questions about provision of free essential medicines and because pregnant women's drug treatment is not captured by the drug use indicators
Provision of essential medicines free at the point of care to elderly patients	Excluded because of similarity to other questions about provision of free essential medicines and because elderly patients' drug treatment is not captured by the drug use indicators
**Regulatory policies**	
Antibiotics not available over-the-counter	Included but graded response adapted to yes/no response as follows: never/occasionally available = yes; always/frequently available = no
Injections not available over-the-counter	Included but graded response adapted to yes/no response as follows: never/occasionally available = yes; always/frequently available = no
Active monitoring of adverse drug reactions	Included
Joint regulation of drug promotion by government and industry (as opposed to regulation by government alone)	Included
**Structural policies**	
National MOH unit promoting rational use of medicines	Included
MOH mandate to have drug and therapeutics committees	Excluded because it is too similar to the other DTC variables, which capture actual policy better and because a mandate was difficult to define
Half or more of all referral hospitals have a DTC	Excluded since the prescribing indicators relate to primary care in primary care centres and general hospitals, but not referral hospitals
Half or more of all general hospitals have a DTC	Included but graded response adapted to yes/no response as follows: all/most/half = yes; few/none = no
Half or more of all provinces/districts have a DTC	Included but graded response adapted to yes/no response as follows: all/most/half = yes; few/none = no
National reference laboratory for antimicrobial resistance	Excluded because of similarity to other “AMR” questions
National task force to contain antimicrobial resistance	Excluded because insufficient numbers of countries responded
Presence of national drug information centre	Included
**Human resource policies**	
Prescribing by doctors in public primary care	Included but graded response adapted to yes/no response as follows: always = yes; frequently/occasionally/never = no
Prescribing by nurses in public primary care	Included but graded response adapted to yes/no response as follows: always/frequently = yes; occasionally/never = no
Prescribing by pharmacists in public primary care	Excluded because pharmacists do not generally determine prescribing
No prescribing by untrained staff (i.e., with less than 1 month's training) in public primary care	Included but graded response adapted to yes/no response as follows: never = yes; always/frequently/occasionally = no

*Includes all the policy questions hypothesized to act on the QUM that were asked of ministries of health in the surveys of 2003 and 2007 [12],[13].

DTC, drug and therapeutics committee; MOH, ministry of health; NMP, national medicines policy.

#### Quality use of medicines indicators (Table 2)

QUM indicators were drawn from those developed and validated by the INRUD [20] and IMCI programmes [21]. We excluded indicators that measured other aspects of patient care or health system functioning. The indicators, with their specified directions of “better” or “worse” use of medicines, are described in Table 2. For example, antibiotics are generally not recommended for acute upper respiratory infections, which are mostly viral in nature, so lower rates of prescribing were categorised as better use. We also excluded a small number of indicators that were not expressed as proportions and indicators where data were sparse, i.e., for which fewer than five countries reported data. This left ten indicators for inclusion in the analyses (Table 2).

**Table 2:** Medicines use indicators and direction of better use.

Variable Name	Direction of Better Use
Percent of patients prescribed antibiotics	Less
Percent of acute diarrhoea cases treated with anti-diarrhoeal drugs	Less
Percent of acute diarrhoea cases treated with antibiotics	Less
Percent of acute diarrhoea cases treated with oral rehydration solution	More
Percent of prescribed drugs belonging to the essential medicines list	More
Percent of drugs prescribed by generic name	More
Percent of patients prescribed injections	Less
Percent of acute pneumonia cases treated with an appropriate antibiotic	More
Percent of patients treated in compliance with guidelines	More
Percent of acute upper respiratory tract infection cases treated with antibiotics	Less

Ten standard medicine use indicators [20],[21] expressed as proportions and reported in surveys in more than four countries during 2002–2008.

#### Measures of national wealth

We extracted data on gross national income per capita (GNIpc) for each country in 2009 from the World Bank (http://data.worldbank.org/indicator/NY.GDP.PCAP.CD).

### Analyses

#### Univariate analyses

**Figure 1:**
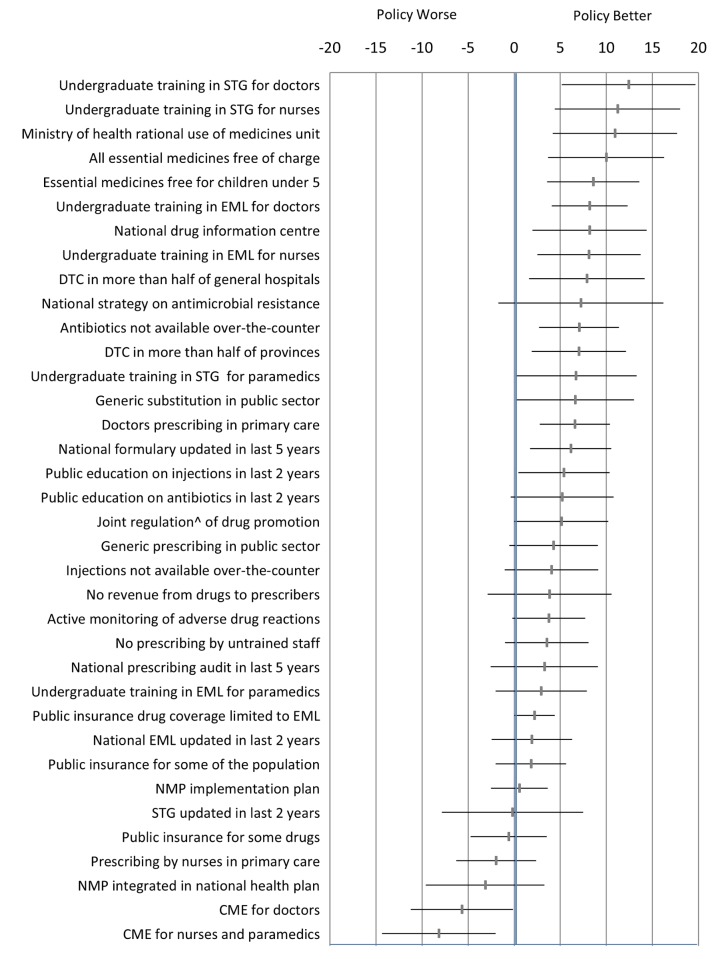
Differences in quality use of medicines between countries that did versus did not report implementation of specific medicine policies. The figure presents the weighted mean (and 95% confidence interval) of differences (in percent) across ten selected QUM measures. ^∧^Joint regulation by government and industry as opposed to government regulation only. CME, continuing medical education; DTC, drug and therapeutics committee; EML, essential medicines list; NMP, national medicines policy; STG, standard treatment guidelines.

Each policy was the principal unit of analysis. We ensured that the directionality of “improved” or “worse” was aligned for each of the ten QUM indicators. For each QUM indicator we calculated the mean difference (as percent), with its standard error, between countries reporting implementation of that policy versus not. These differences were averaged to give an overall difference across the indicators for each policy. The difference could be positive or negative. We calculated the weighted mean difference to account for the fact that varying numbers of countries reported data on specific QUM indicators. The estimated differences, with their 95% confidence intervals, are given in Figure 1 and represent our best estimates of the quantitative impacts of the individual policies. We did not perform direct “head to head” comparisons of the impacts of the different policies and did not adjust the statistical analyses for multiple comparisons.

#### Correlations of multiple policies with a composite quality use of medicines variable

We realised that countries employed various combinations of policies, but there was too much heterogeneity and insufficient sample size to measure the joint effects of particular combinations. So we looked for correlations between the numbers of implemented policies (derived from those deemed effective in the univariate analyses) and a composite score of QUM. Countries were the unit of analysis, and all countries were given equal weight in the regression analyses. We derived the policy implementation and QUM variables as follows. We estimated the number of policies that a country reported implementing, out of the total number of 27 policies that were associated with effect sizes of 2% or more, and separately for the 18 policies that were associated with effect sizes significantly (unadjusted *p*<0.05) different from zero (Figure 1). Since a number of countries did not respond on whether they implemented a specific policy, the variable had to be adjusted for missing data. We used the following formula: 
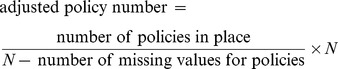
(1)where *N* was 27 or 18. Countries typically had survey data for only a subset of the ten medicine use indicators. Therefore, we created a composite measure of QUM to enable all 56 countries to be included in the analyses. For each country and each individual QUM variable, we calculated how far that country lay above or below the mean value from all countries reporting values for that variable. We used standard deviation units, so the number was dimensionless. We calculated the mean value (in standard deviation units) for each country (which could be positive or negative) across the multiple medicine use measures, and this was regressed on two “number of policies” variables: (1) the number out of 27 policies with an effect size of 2% or more, estimated from the univariate analyses (27-policy variable) and (2) the number out of 18 policies with an effect size that was statistically significantly (unadjusted *p<*0.05) different from zero (18-policy variable). We assessed the impact of national wealth in two ways. We included each country's value for GNIpc in multiple linear regressions of the composite QUM measure on each of the two number-of-policies variables. We also performed simple linear regression analysis of the composite QUM measure on the 27-policy and 18-policy variables separately for countries that had GNIpc values above or below the median for the whole group (US$2,333). In addition, we included the GNIpc values in the regressions of the QUM scores on the two number-of-policies variables in countries with wealth levels above and below the median value. Finally, we regressed the values for two selected individual medicine use indicators (percentage of acute upper respiratory tract infection cases receiving antibiotics; percentage of acute diarrhoea cases receiving oral rehydration solution) on the policy variables. These were the indicators reported by the greatest number of countries; they have the advantage that they are widely valued measures of quality of care and enabled estimation of the health impacts of policies that were not apparent from standard deviation units. We repeated all regression analyses using a non-parametric approach (Spearman rank correlation). All analyses were done with StatsDirect (version 2.7.9; StatsDirect).

## Results

Sixty-four developing and transitional countries were identified in the WHO database with analysable data on the ten selected QUM indicators, of which 56 also had analysable data on policies. On average, for the period 2002–2008, there were three QUM surveys per country, and data on each of the ten QUM indicators was collected (between all the surveys done) at least once per country (Table 3). Out of a potential 2,016 policy responses (36 policies in each of 56 countries), 1,646 (82%, 95% CI 80% to 83%) were available. Of 56 countries with relevant policy and QUM data, 24 countries provided policy data in both 2003 and 2007. Out of a potential 864 policy responses (24 countries ×36 policies), 174 responses (20%, 95% CI 18% to 23%) differed between 2003 and 2007, of which 123 responses, or 14% (95% CI 12% to 17%) of all responses from the 24 countries reporting policy data in 2003 and 2007, were excluded from the analysis. The regional distribution of the 56 countries was Africa, 24; Eastern Mediterranean, 9; Europe, 4; Latin America, 7; South East Asia, 4; and Western Pacific, 8.

**Table 3:** Results for the ten individual medicine use measurements derived from surveys conducted in the 56 study countries.

Country	Percent of Patients Treated with Antibiotics	Percent of Acute Diarrhoea Cases Treated with Anti-Diarrhoeal Agents	Percent of Acute Diarrhoeal Cases Treated with Antibiotics	Percent of Acute Diarrhoea Cases Treated with Oral Rehydration Solution	Percent of Prescribed Drugs Belonging to the Essential Drug List	Percent of Drugs Prescribed by Generic Name	Percent of Patients Treated with Injections	Percent of Acute Pneumonia Cases Treated with the Correct Antibiotic	Percent of Patients Treated in Compliance with Standard Treatment Guidelines	Percent of Acute Upper Respiratory Tract Infection Cases Treated with Antibiotics
Armenia				86.00					59.00	
Angola	38.20	0.30	37.60	41.20	58.80	60.80	4.60	39.20		49.00
Bahrain	27.60									
Bolivia									39.30	
Brazil	28.80	5.00	24.30	90.00	79.40	83.70	6.70	49.30	51.40	67.25
Burkina Faso	58.00			44.00		93.00	23.00		34.00	
Burundi	50.00	0.00	60.00	40.00	92.40	87.30	10.00	90.00		90.00
Cambodia	60.00	0.00	60.00	100.00	100.00	100.00	57.60	100.00	89.10	0.00
Cameroon	62.90	10.00	75.00	77.50	92.50	88.80	45.00	80.00		100.00
Chile								83.10		
China	55.23	45.00	52.50	36.63	80.25		44.91			75.59
Colombia	30.00	11.00	53.00	49.00	94.20		13.30	37.00		54.00
Congo	43.40	80.00	65.00	10.00	59.10	57.00	40.00	95.00		100.00
Cuba								93.30		71.50
Democratic Republic of the Congo	66.00	0.00	60.00	80.00	88.00	54.90	31.60	60.00		100.00
Egypt								72.00	74.00	
Ethiopia	56.80	4.66	51.81	72.80			21.81	52.49	43.00	58.06
Gambia	50.00	0.00	70.00	70.00	100.00	69.60	28.40			90.00
Ghana	42.80	0.00	53.10	76.60	93.90		34.90	91.10		81.20
Guatemala	41.50				97.00		10.50			
Guinea	64.50	1.00	66.00	86.00				68.00		93.00
India	50.77		77.80		91.56	44.43	18.69			75.70
Indonesia	50.00	5.00	55.00	95.00	100.00		0.00	100.00	47.40	20.00
Iran	70.00				100.00		30.00			
Jordan	61.50							89.20		84.90
Kenya	73.36	2.76	53.21	54.45	79.28	48.57	34.14	67.27	15.75	73.77
Kyrgyzstan	35.00				59.00		30.00			
Lao People's Democratic Republic	47.00	5.00	46.00	77.00	86.15	75.85	18.00	91.00	74.00	41.00
Malawi			49.00	55.33				61.00	55.33	77.00
Malaysia	21.73				100.00	42.05	0.00			29.80
Mali	58.90	34.70	77.70	46.80	94.65	93.30	35.15	60.50	23.16	91.50
Mongolia	27.40								15.40	
Morocco				83.00			1.00	72.00	30.90	
Mozambique			34.00	93.00				97.00		55.00
Namibia	50.50					65.50				
Nepal	50.65	40.00	55.35	45.75	74.93	58.97	5.60	79.10	18.10	46.20
Niger				8.00				76.00	21.00	
Nigeria	53.50	40.00	80.00	40.00	86.80	41.80	10.95	40.00		100.00
Oman	38.63	6.50	19.50	91.50	97.50		6.39			52.00
Pakistan	55.20						37.10			
Peru	59.00				72.00	70.00	26.00		76.00	
Philippines	55.40				58.60		0.00		42.90	
Rwanda	50.00	0.00	60.00	70.00	94.00	80.00	20.00	90.00		90.00
Samoa	56.00				75.00	28.00	18.00			
Senegal	46.00	25.70	34.95	61.60	83.20	60.00	25.00	56.65	78.50	79.30
Serbia and Montenegro	27.50									
South Africa	27.00					59.50		64.20	32.07	
Sudan	70.40		61.97	47.51	86.08	27.98	26.80	44.40	50.42	
Thailand										60.40
Tonga	53.50	2.00	17.35	67.00	99.00	37.00	7.00	84.50		65.00
Tunisia	55.50									
Uganda	64.30	3.70	49.75	68.05	92.15	71.50	23.10	45.00	49.00	94.15
Tanzania	46.43	5.00	44.00	71.00	98.50	87.45	25.67	86.50	68.35	87.50
Uzbekistan				0.00				0.00	38.89	
Viet Nam								33.30	33.30	
Zambia	59.00		30.00	61.00	100.00	41.00	16.00	86.80	84.45	90.00

The number of completed measures of medicine use varied by country. The impact of incomplete data on the results of the regression analyses of the composite measure of medicine use on number of policies reported as implemented was explored in sensitivity analyses (see Results).

### Estimating the Impacts of Individual Essential Medicines Policies

Information on the self-reported implementation of the 36 individual essential medicines policies by the 56 study countries is provided in Table S1. Table 3 lists the survey-derived values for the ten key QUM indicators for these countries. Additional information, including references for the studies from which the medicine use measures were obtained and the GNIpc figures for each country, are provided in Table S2. Interpretation of the medicine use data requires specification of whether higher values reflect better or worse care, and this is provided in Table 2.

It will be appreciated from Table 3 that there were substantial missing outcomes data, and the impact of this on the regression analyses is tested in sensitivity analyses below.

Figure 1 and Table 4 summarise the weighted mean differences (as percent) across ten QUM indicators between countries that did versus did not report implementation of specific policies. Twenty-seven policies were associated with effect sizes of 2% or more, and 18 policies demonstrated unadjusted *p*-values of less than 0.05 for association with better medicine use. The largest positive associations were seen with undergraduate training in standard treatment guidelines for doctors (12.4%, 95% CI 5.2% to 19.7%) and nurses (11.2%, 95% CI 4.4% to 18.0%), the ministry of health having a unit promoting rational use of medicines (10.9%, 95% CI 4.2% to 17.7%), and provision of essential medicines free at point of care to all patients (10.0%, 95% CI 3.6% to 16.3%) and to children under 5 y of age (8.6%, 95% CI 3.6% to 13.6%). Negative effects were associated with continuing medical education for doctors (−5.7%, 95% CI −0.1% to −11.2%) and for nurses and paramedical workers (−8%, 95% CI −2.0% to −14.3%).

**Table 4:** Differences in quality use of medicines between countries that did versus did not report implementation of specific medicine policies.

Policy	Weighted Mean Difference (Percent)	95% Confidence Interval
Undergraduate training in STG for doctors	12.4	5.2 to 19.7
Undergraduate training in STG for nurses	11.2	4.4 to 18.0
Ministry of health unit promoting rational use of medicines	10.9	4.2 to 17.7
Essential medicines free at the point of care for all patients	10.0	3.7 to 16.3
Essential medicines free at the point of care for children under 5 y	8.6	3.6 to 13.6
Undergraduate training in EML for doctors	8.2	4.1 to 12.3
National drug information centre	8.2	2.0 to 14.4
Undergraduate training in EML for nurses	8.1	2.5 to 13.7
DTC in more than half of general hospitals	7.9	1.6 to 14.2
National strategy on antimicrobial resistance	7.2	−1.7 to 16.2
Antibiotics not available over-the-counter	7.0	2.7 to 11.4
DTC in more than half of provinces	7.0	1.9 to 12.1
Undergraduate training in STG for paramedics	6.7	0.2 to 13.3
Generic substitution in public sector	6.6	0.3 to 13.0
Prescribing by doctors in primary care	6.6	2.8 to 10.4
National formulary updated in last 5 y	6.1	1.7 to 10.5
Public education on injections in last 2 y	5.4	0.4 to 10.3
Public education on antibiotics in last 2 y	5.2	−0.4 to 10.8
Joint regulation∧ of drug promotion	5.1	0.01 to 10.2
Generic prescribing in public sector	4.3	−0.5 to 9.1
Injections not available over-the-counter	4.1	−1.0 to 9.1
No revenue from drugs to prescribers	3.8	−2.9 to 10.6
Active monitoring of adverse drug reactions	3.8	−0.2 to 7.7
No prescribing by untrained staff	3.5	−1.0 to 8.1
National prescribing audit in last 5 y	3.3	−2.6 to 9.1
Undergraduate training in EML for paramedics	2.9	−2.0 to 7.9
Public insurance drug coverage limited to EML	2.2	0.03 to 4.4
National EML updated in last 2 y	1.9	−2.5 to 6.3
Public insurance for some of the population	1.8	−2.0 to 5.7
NMP implementation plan	0.6	−2.5 to 3.7
STG updated in last 2 y	−0.2	−7.9 to 7.5
Public insurance for some drugs	−0.6	−4.7 to 3.5
Prescribing by nurses in primary care	−2.0	−6.3 to 2.4
NMP integrated into national health plan	−3.1	−9.6 to 3.3
CME for doctors	−5.7	−11.2 to −0.1
CME for nurses and paramedics	−8.2	−14.3 to −2.0

The table presents the weighted mean (and 95% confidence interval) of differences (in percent) across ten selected measures of QUM between countries that did versus did not report implementation of specific medicine policies.

∧Joint regulation by government and industry, as compared to regulation by government alone.

CME, continuing medical education; DTC, drug and therapeutics committee; EML, essential medicines list; NMP, national medicines policy; STG, standard treatment guidelines.

### Effects of Multiple Policies and Impact of National Wealth

There were weak to moderate positive correlations between the numbers of essential medicines policies reported implemented and the composite QUM indicator: *r* = 0.39 (95% CI 0.14 to 0.59), *p = *0.003, for the 27-policy variable, and *r* = 0.39 (95% CI 0.14 to 0.59), *p = *0.003, for the 18-policy variable. In order to assess the impact of missing QUM data (Table 3), we repeated the regression analyses including only countries with three or more, or five or more, observations contributing to their composite QUM score. This generated stronger correlations than were seen in the base case: *r* = 0.48 (95% CI 0.21 to 0.68), *p = *0.001, for three or more observations, and *r* = 0.64 (95% CI 0.38 to 0.81), *p<*0.0001, for five or more observations (Figure S7).

There were weak to moderate correlations between per capita national wealth (GNIpc) and the QUM indicator (*r* = 0.51, 95% CI 0.28 to 0.68, *p*<0.001) and between GNIpc and the numbers of policies implemented: 27-policy variable, *r* = 0.36 (95% CI 0.11 to 0.57), *p* = 0.006; 18-policy variable, *r* = 0.30 (95% CI 0.04 to 0.52), *p* = 0.024.

To adjust for confounding by national wealth levels, we performed multiple linear regression analyses that included the number-of-policies variables and GNIpc. In these analyses the regression coefficients for the number-of-policies variables weakened (*r* = 0.25, 95% CI −0.015 to 0.52, *p = *0.064, for the 27-policy variable, and *r* = 0. 29, 95% CI 0.025 to 0.56, *p = *0.033, for the 18-policy variable) but remained statistically significant for the 18-policy variable.

**Figure 2:**
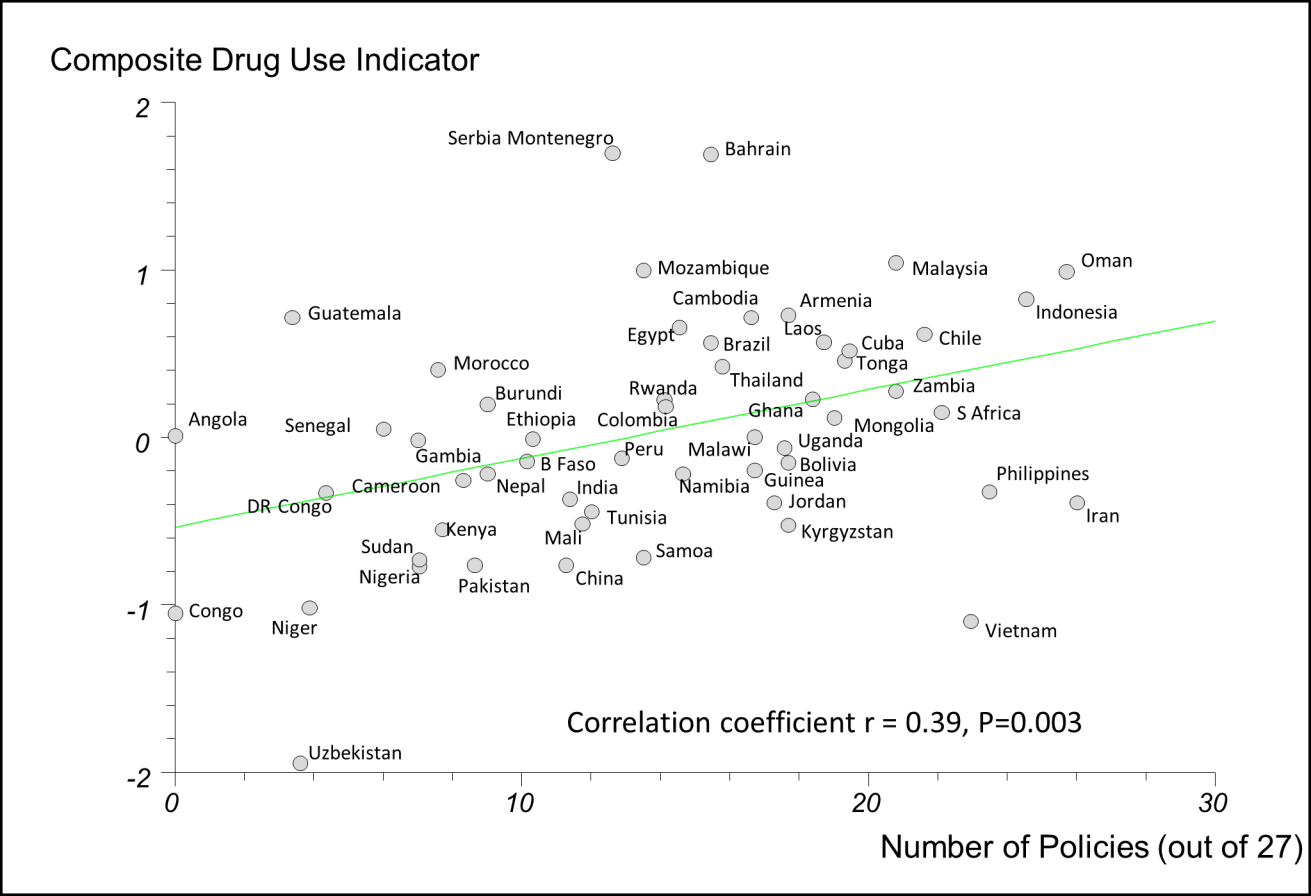
Correlation between the number of policies that countries reported implementing (out of 27) and a composite measure of quality use of medicines in 56 countries. Details of calculation of the composite QUM measure are provided in the Methods. B Faso, Burkina Faso; DR Congo, Democratic Republic of the Congo; Laos, Lao People's Democratic Republic; S Africa, South Africa; Serbia Montenegro, Serbia and Montenegro.

Figure 2 shows the regression analysis for QUM versus the 27-policy variable, with results for individual countries highlighted. The figures illustrating the other regression analyses referred to here are provided as Figures S1–S6.

We performed separate regression analyses using the 27-policy variable in countries with national wealth measures above and below the median value for GNIpc (US$2,333). In countries with GNIpc above the median value, the correlation coefficient was weak and not statistically significant: *r* = 0.22 (95% CI −0.15 to 0.56), *p = *0.261(Figure S5). In countries with GNIpc values below the median, the correlation was moderate and statistically significant: *r* = 0.43 (95% CI 0.06 to 0.69), *p = *0.023 (Figure S6). Importantly, the inclusion of GNIpc in a multiple linear regression analysis did not weaken the association of multiple policies with better QUM in the lower-income countries: *r* = 0.44 (95% CI 0.075 to 0.81), *p = *0.020 (27-policy variable).

**Figure 3:**
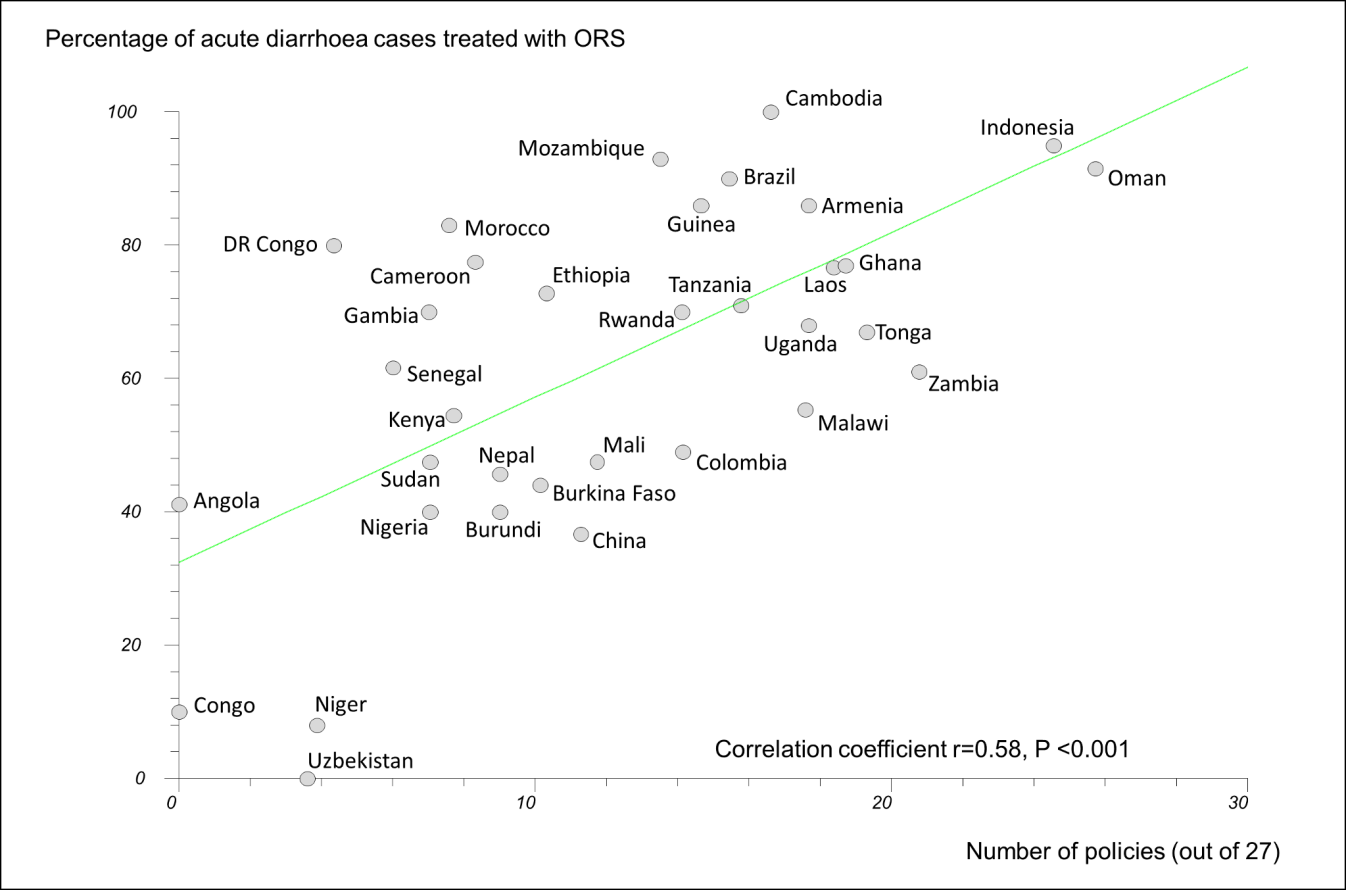
Correlation between number of implemented policies and percentage of cases of acute diarrhoeal illness treated with oral rehydration solution. DR Congo, Democratic Republic of the Congo; Laos, Lao People's Democratic Republic; ORS, oral rehydration solution.

**Figure 4:**
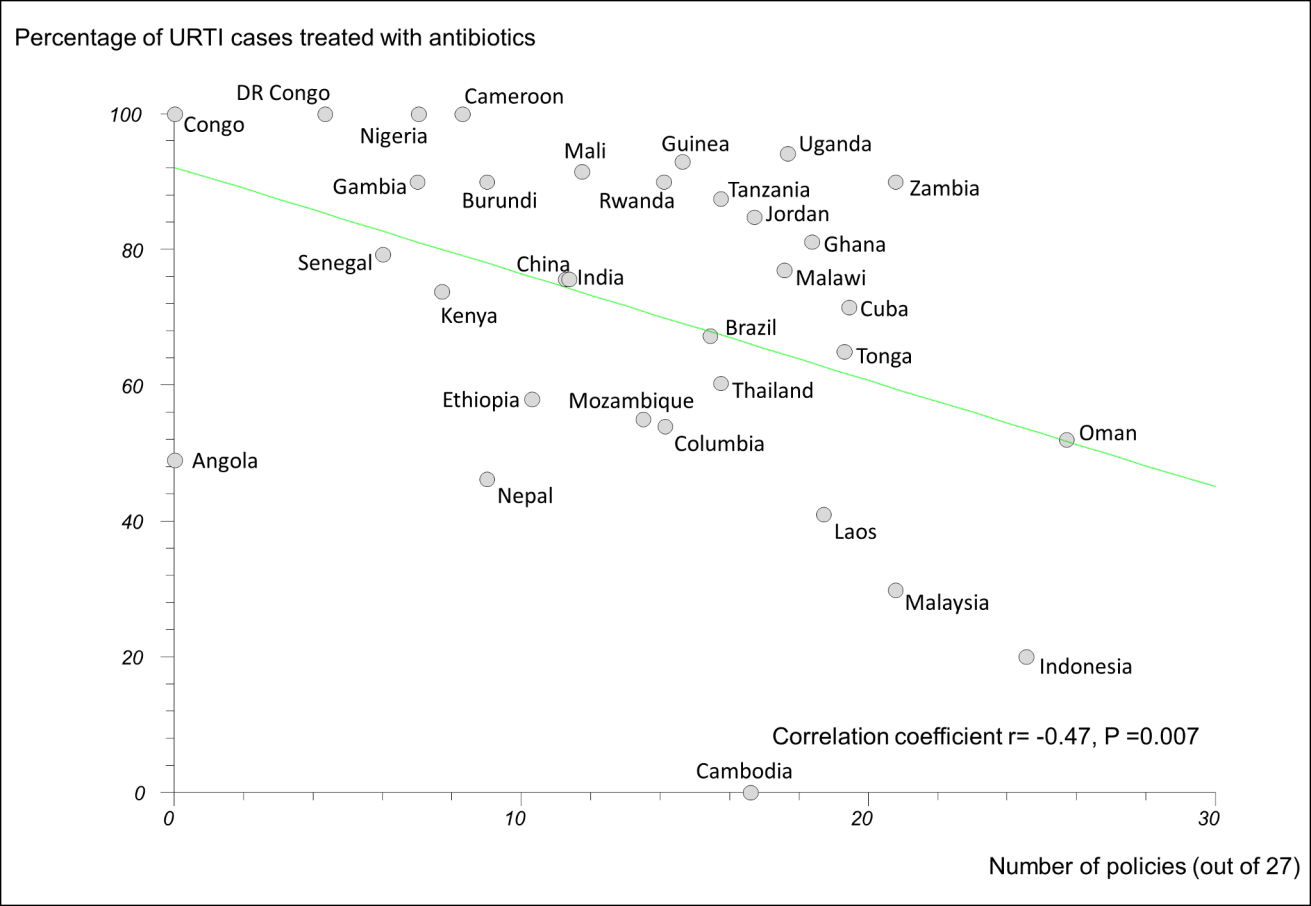
Correlation between number of implemented policies and percentage of cases of acute upper respiratory tract infection treated with antibiotics. DR Congo, Democratic Republic of the Congo; Laos, Lao People's Democratic Republic; URTI, acute upper respiratory tract infection.

In regression analyses using the 27-policy variable, the number of medicine policies correlated positively (as expected) with use of oral rehydration solution in acute diarrhoeal illness (*r* = 0.58, 95% CI 0.29 to 0.77, *p*<0.001) (Figure 3) and negatively (as expected) with use of antibiotics in upper respiratory infection (*r* = −0.47, 95% CI −0.71 to −0.14, *p = *0.007) (Figure 4).

For the main analyses, non-parametric regression provided results similar to those presented here (Table S3).

## Discussion

Our most important findings were 2-fold. First, some essential medicines policies—particularly the provision of undergraduate training in standard treatment guidelines to doctors and nurses, provision of essential medicines free at point of care to all patients, and having a ministry of health unit promoting QUM—were associated with improved measures of QUM in a multi-country study. Second, there was a positive correlation between the number of medicine policies that countries reported implementing and the quality of their medicine use. This correlation was strongest and statistically significant in countries with national per capita wealth levels below the median of the study countries (US$2,333), underscoring the importance of essential medicines policies in low-income countries.

We found that national wealth was associated with a larger number of implemented policies and also with better medicine use. Consequently, we treated wealth as a confounder in our analyses of the impacts of policies. Wealth's importance has been reported previously but, to our knowledge, has not been quantified in a multi-country analysis [3],[12],[13]. It may be that economic status improves QUM through generally better health systems and more educated and healthy populations. However, it could also be that increased economic status enables policy implementation and that it is the policies themselves that lead to better medicine use. The finding that the correlation between number of policies reported implemented and QUM was unaffected by inclusion of GNIpc in a multi-linear regression analysis in low-income countries suggests that the policies may be the most important factor. The findings of this study are in agreement with several literature reviews that point to the importance of multiple policy interventions [3],[14],[15],[25]–[27], but this is the first time, to our knowledge, that this approach has been shown to be important on a global scale.

While the data presented here cannot prove causality, they provide the strongest evidence to date that some essential medicines policies are effective. The benefits of multiple policies were seen via a composite measure of QUM that incorporated ten indicators, as well as by variables that reflected optimal treatment of acute respiratory tract infection and acute diarrhoeal illness, strategies that have been shown to lead to improved health outcomes. The associations of the policies with these two conditions were robust and comparable with the intervention effects reported elsewhere [3],[14],[15]. We believe the sustainability of such improvements in treatment will be greater with national policy implementation than with the discrete interventions that are reported in most reviews [3],[14],[15],[26],[27].

### Comparisons with Previous Studies

Our findings are consistent with the literature, which shows that educational interventions are associated with improved prescribing [3],[14],[15], but the effects are limited and short term unless there is follow-up [1]. Published studies also show that some managerial interventions are effective [1],[3],[14],[15]. This was confirmed here for provision of free essential medicines, establishment of drug and therapeutics committees, non-availability of antibiotics over-the-counter, and several others. Studies point to the effects (positive and negative) of other policies we examined. A national ministry of health unit to promote rational use of medicines was associated with improved medicine use in Oman [28]; enforcing non-availability of antibiotics over-the-counter in Chile was associated with reduced antibiotic use [29]; public education resulted in modestly improved antibiotic use in Europe [17]. Supplementation of prescriber income by the selling of medicines has been seen to be associated with worse medicine use in China [30] and Zimbabwe [31],[32], and separation of the prescriber and dispenser functions in Korea was associated with reduced inappropriate antibiotic use [33]. Government regulation of drug promotion has been found to be more effective than self-regulation [34]. In our study, joint government and industry regulation was associated with better medicine use than government regulation alone.

It is important to note that other potentially effective policies were not examined in this study. For example, prescription audit and feedback and some pricing policies have been found effective [3],[14],[15] and form part of a broader range of policy instruments that should be considered by governments.

Disconcertingly, policies encouraging continuing medical education of doctors, nurses, and paramedical workers were associated with actual worsening of QUM. In our view, this may be because such activities in the study countries were funded by the pharmaceutical industry. The effectiveness of continuing medical education conducted in other circumstances may be different.

### Potential Sources of Bias

Our findings should be interpreted carefully. Governments with effective overall health policies may be more likely to implement rational medicine policies; consequently, it may be the general functioning of the health care system in these countries that leads to better QUM. Some outcomes may be a result of specific vertical disease policies. There was a moderate to strong positive correlation between the number of medicine policies reported as implemented and the frequency of oral rehydration solution use in acute diarrhoea. This may be because governments that are successful in implementing acute diarrhoea treatment programmes are those that also implement essential medicines policies. On the other hand, the moderate to strong inverse correlation between the number of policies reported as implemented and the use of antibiotics in acute upper respiratory tract infection is more likely to point to a specific effect of the medicine policies.

### Limitations

The study design is, in effect, an ecological study using secondary data analysis showing that the more policies that are implemented, the better the QUM. However, without consistent longitudinal data (which do not exist in many countries), causality cannot be inferred.

The policy data are based on responses to questionnaires sent to ministries of health, and there may be an incentive to over-report successes in policy implementation or to report that policies were implemented when, in fact, they were not. If this happened, then the associations between policy implementation and QUM that we found are likely to be weaker than if policy implementation had been reported correctly. Furthermore, the assumption that policies remained the same throughout 2002–2008 for those countries not reporting in both 2003 and 2007 may not be correct. Nevertheless, the fact that there was only a 20% difference in reported policies for those countries reporting in both 2003 and 2007 suggests that the majority of policies are likely to have remained the same throughout the period.

We were also reliant on surveys to measure QUM. These were not part of a single planned prospective study. However, the WHO, in collaboration with INRUD, did extensive work to establish minimum standards (methodology and indicators) for surveying medicine use in primary health care facilities [3],[14]. Consequently, the surveys we relied on had standardised methods and met minimum sample size recommendations. While some surveys may not have produced outcome data generalisable to the whole country, any inaccuracies of drug use estimates would tend to weaken the associations we found.

Of greater concern would be biased recording of results, i.e., if those countries that implemented more policies selectively reported positive outcomes. We believe this is not likely, as the data on policy implementation and drug use came from independent sources. The former were submitted by ministries of health, and the latter were derived from the publications of independently conducted surveys.

It should be noted that there is a further limitation with both the reported policy implementation and medicine use data in that while these data are used successfully here to show correlation between reported policy implementation and better medicine use, they cannot be used to compare or benchmark the performance of individual countries.

Many of the comparisons involved small sample sizes. For this reason, a composite indicator that captured several dimensions of QUM was created. This increased the effective sample size, and enabled correlations between multiple policies and QUM to be tested. However, the clinical relevance of a change in a composite measure is unclear. Consequently, we analysed two measures of actual QUM, namely, inappropriate use of antibiotics in acute upper respiratory tract infection and appropriate use of oral rehydration solution in acute diarrhoea. These analyses showed substantially better medicine use in countries implementing more policies.

Our results were confined to the public sector. Essential medicines policies seek to influence QUM in both the public and private sector. Unfortunately, there were insufficient data on QUM in the private sector. In many low- and middle-income countries, the majority of health care is provided by the private sector, where QUM has been found to be worse than in the public sector, partly because of perverse financial incentives [3],[14],[15]. The absence of private sector data in this study does not diminish the significance of our findings. Policies aimed at the public sector may also impact on the private sector since many prescribers work in both sectors. Furthermore, some low- and middle-income countries are looking to expand the public sector and introduce insurance programmes with medicine coverage. This study provides the first evidence, to our knowledge, to enable the selection of policies that are likely to have the biggest impact.

In conclusion, our findings indicate that countries reporting implementation of multiple essential medicines policies have better QUM than those that do not. This association is strongest in low-income countries. International support for the WHO, increasingly, is being channelled to vertical disease programmes (e.g., addressing AIDS, tuberculosis, and malaria) and away from horizontal programmes designed to support development and maintenance of health policies and standards. The medicine policies and data discussed here were developed and collected as part of the WHO core “normative” functions, which are now under threat [35],[36]. It is important that the critical role of the WHO is recognised and that these efforts are sustained and enhanced.

## Supporting Information

Figure S1 Correlation between the number of policies that countries reported implementing (out of 18) and a composite measure of quality use of medicines in 56 countries.
(DOCX)

Figure S2 Correlation between gross national income per capita and a composite measure of quality use of medicine in 56 countries.
(DOCX)

Figure S3 Correlation between gross national income per capita and the number of policies countries report implementing (27-policy variable).
(DOCX)

Figure S4 Correlation between gross national income per capita and the number of policies countries report implementing (18-policy variable).
(DOCX)

Figure S5 Correlation between the number of policies that countries reported implementing (out of 27) and a composite measure of quality use of medicines in countries with gross national income per capita values above the median for the group (US$2,333).
(DOCX)

Figure S6 Correlation between the number of policies that countries reported implementing (out of 27) and a composite measure of quality use of medicines in countries with gross national income per capita values below the median for the group (US$2,333).
(DOCX)

Figure S7 Correlation between the number of policies that countries reported implementing (out of 27) and a composite measure of quality use of medicines in 56 countries. This analysis was restricted to countries that had five or more measures of medicine use contributing to their composite QUM score.
(DOCX)

Table S1 Reported implementation of policies by country.
(XLSX)

Table S2 Individual medicine use measures by country and with references.
(XLS)

Table S3 Spearman rank correlation coefficients for the main regression analyses.
(DOCX)

## Abbreviations

GNIpc: gross national income per capita
IMCI: Integrated Management of Childhood Illness
INRUD: International Network for the Rational Use of Drugs
QUM: quality use of medicines
WHO: World Health Organization
