# Supplementary material for: WHO Essential Medicines Policies and Use in Developing and Transitional Countries: An Analysis of Reported Policy Implementation and Medicines Use Surveys
Source: PLoS Med. 2014 Sep 16;11(9):e1001724. doi: 10.1371/journal.pmed.1001724 (PMC4165598; doi:10.1371/journal.pmed.1001724)

**Supporting Information Figure S1**

Correlation between the numbers policies that countries reported implementing (out of 18) and a composite measure of quality use of medicines in 56 countries.


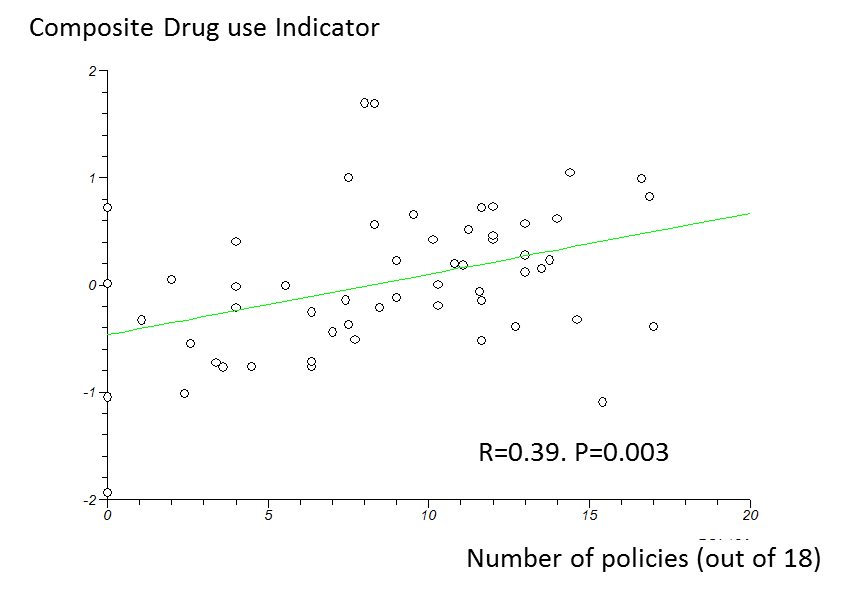

Supplement: Figure S1 — Correlation between the number of policies that countries reported implementing (out of 18) and a composite measure of quality use of medicines in 56 countries. (DOCX) [file pmed.1001724.s001.docx]
