# Supplementary material for: WHO Essential Medicines Policies and Use in Developing and Transitional Countries: An Analysis of Reported Policy Implementation and Medicines Use Surveys
Source: PLoS Med. 2014 Sep 16;11(9):e1001724. doi: 10.1371/journal.pmed.1001724 (PMC4165598; doi:10.1371/journal.pmed.1001724)

**Supporting Information Figure S2**

Correlation between gross national income per capita (GNIpc) and a composite measure of quality use of medicine in 56 countries


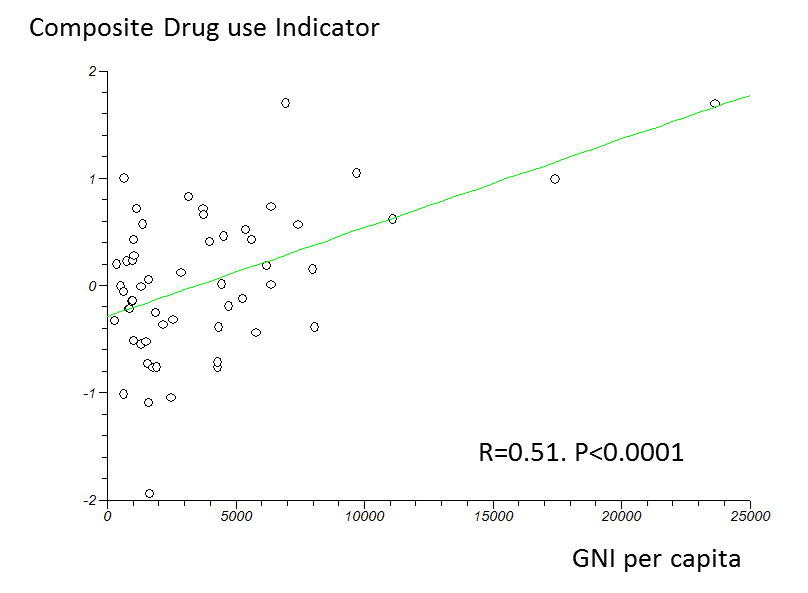

Supplement: Figure S2 — Correlation between gross national income per capita and a composite measure of quality use of medicine in 56 countries. (DOCX) [file pmed.1001724.s002.docx]
