# Supplementary material for: WHO Essential Medicines Policies and Use in Developing and Transitional Countries: An Analysis of Reported Policy Implementation and Medicines Use Surveys
Source: PLoS Med. 2014 Sep 16;11(9):e1001724. doi: 10.1371/journal.pmed.1001724 (PMC4165598; doi:10.1371/journal.pmed.1001724)

**Supporting Information Figure S3**

Correlation between gross national income per capita (GNIpc) and a the number of policies countries report implementing (27 policy variable)


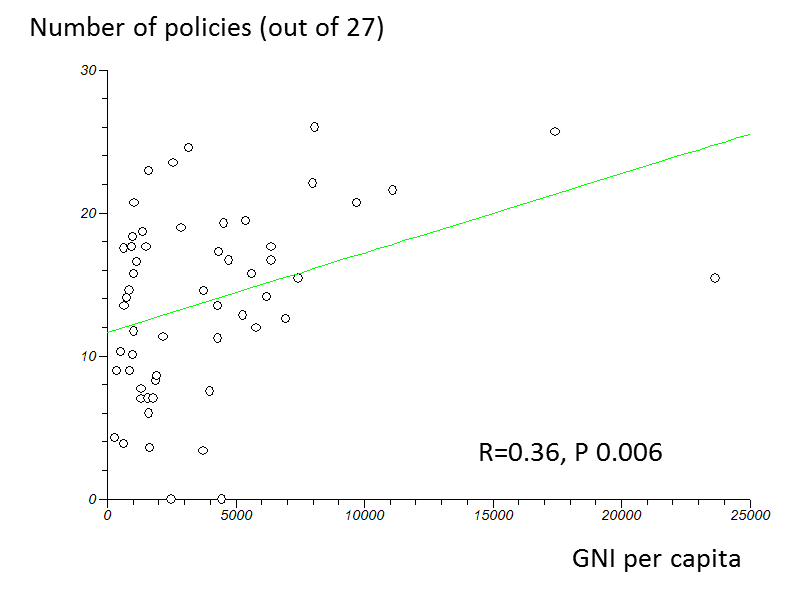

Supplement: Figure S3 — Correlation between gross national income per capita and the number of policies countries report implementing (27-policy variable). (DOCX) [file pmed.1001724.s003.docx]
