# Supplementary material for: WHO Essential Medicines Policies and Use in Developing and Transitional Countries: An Analysis of Reported Policy Implementation and Medicines Use Surveys
Source: PLoS Med. 2014 Sep 16;11(9):e1001724. doi: 10.1371/journal.pmed.1001724 (PMC4165598; doi:10.1371/journal.pmed.1001724)
Supplement: Figure S5 — Correlation between the number of policies that countries reported implementing (out of 27) and a composite measure of quality use of medicines in countries with gross national income per capita values above the median for the group (US$2,333). (DOCX) [file pmed.1001724.s005.docx]

**Supporting Information Figure S5**

Correlation between the numbers policies that countries reported implementing (out of 27) and a composite measure of quality use of medicines in countries with GNIpc values above the median for the group ($2333)

**
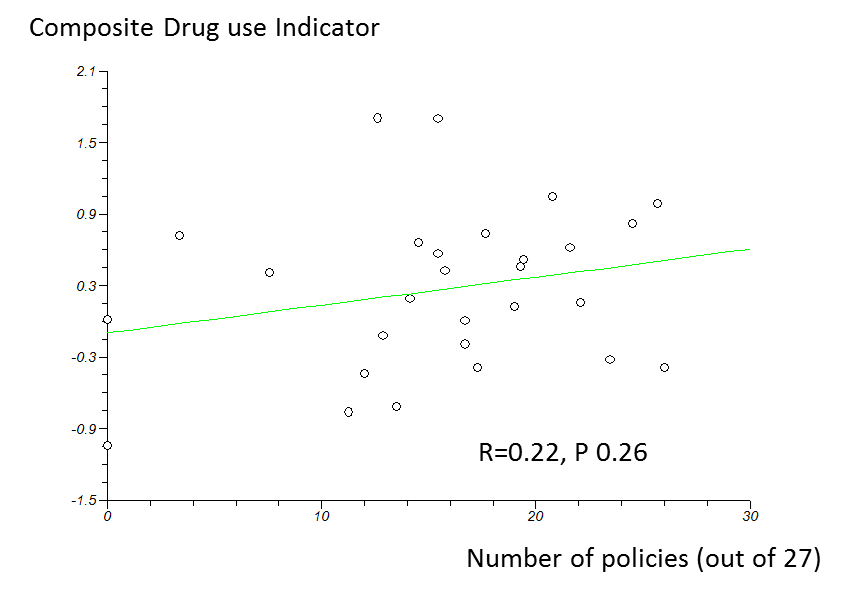
**
