# Supplementary material for: WHO Essential Medicines Policies and Use in Developing and Transitional Countries: An Analysis of Reported Policy Implementation and Medicines Use Surveys
Source: PLoS Med. 2014 Sep 16;11(9):e1001724. doi: 10.1371/journal.pmed.1001724 (PMC4165598; doi:10.1371/journal.pmed.1001724)

**Supporting Information Figure S7**

Correlation between the numbers policies that countries reported implementing (out of 27) and a composite measure of quality use of medicines in 56 countries. This analysis was restricted to countries that had 5 or more measures of medicines use contributing to their composite QUM score


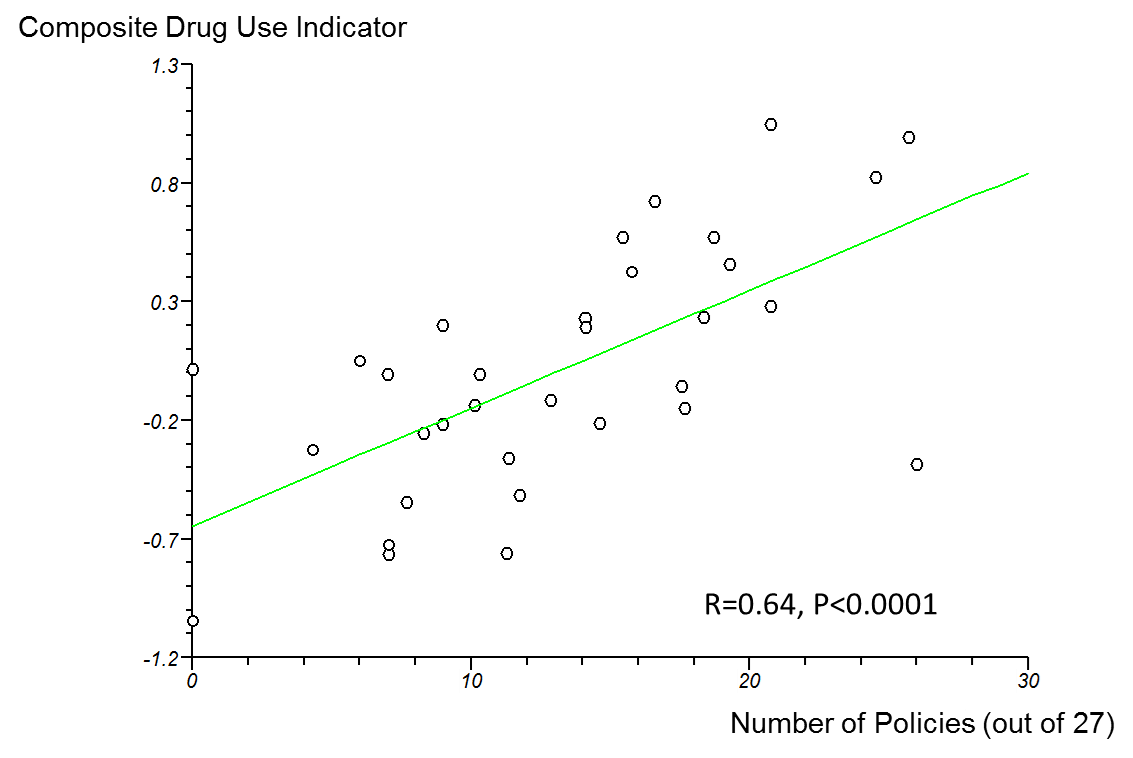

Supplement: Figure S7 — Correlation between the number of policies that countries reported implementing (out of 27) and a composite measure of quality use of medicines in 56 countries. This analysis was restricted to countries that had five or more measures of medicine use contributing to their composite QUM score. (DOCX) [file pmed.1001724.s007.docx]
