# Supplementary material for: WHO Essential Medicines Policies and Use in Developing and Transitional Countries: An Analysis of Reported Policy Implementation and Medicines Use Surveys
Source: PLoS Med. 2014 Sep 16;11(9):e1001724. doi: 10.1371/journal.pmed.1001724 (PMC4165598; doi:10.1371/journal.pmed.1001724)
Supplement: Table S3 — Spearman rank correlation coefficients for the main regression analyses. (DOCX) [file pmed.1001724.s010.docx]

**Supporting information Table S3 (Spearman rank Correlation Coefficients for the**

**main regression analyses**

| Correlation | Spearman’s rank correlation coefficient | 95% LCL | 95% UCL |
| --- | --- | --- | --- |
| Number of policies v composite QUM score (27 policy variable) | 0.39 | 0.14 | 0.59 |
| Number of policies v composite QUM score (18 policy variable) | 0.40 | 0.16 | 0.60 |
| GNIpc v composite QUM score | 0.31 | 0.05 | 0.53 |
| GNIpc v number of policies (27 policy variable) | 0.36 | 0.11 | 0.57 |
| GNIpc v number of policies (18 policy variable) | 0.31 | 0.05 | 0.52 |
| Number of policies v proportion of diarrhea cases treated with oral rehydration solution | 0.49 | 0.17 | 0.71 |
| Number of policies v proportion of upper respiratory infections treated with antibiotics | -0.44 | -0.68 | -0.11 |
